# Inhibition of Stat3 Signaling Pathway by Natural Product Pectolinarigenin Attenuates Breast Cancer Metastasis

**DOI:** 10.3389/fphar.2019.01195

**Published:** 2019-10-10

**Authors:** Yali Li, Cailing Gan, Yange Zhang, Yan Yu, Chen Fan, Yuanle Deng, Qianyu Zhang, Xi Yu, Yiwen Zhang, Liqun Wang, Fang He, Yongmei Xie, Tinghong Ye, Wenya Yin

**Affiliations:** ^1^West China School of Public Health and West China Fourth Hospital and Healthy Food Evaluation Research Center, Sichuan University, Chengdu, China; ^2^Laboratory of Liver Surgery, State Key Laboratory of Biotherapy, West China Hospital, Sichuan University and Collaborative Innovation Center for Biotherapy, Chengdu, China; ^3^Cosmetic Plastic and Burn Surgery, West China Hospital, West China Medical School, Sichuan University, Chengdu, China; ^4^School of Pharmacy, Southwest University for Nationalities, Chengdu, China; ^5^Carey Business School, Johns Hopkins University, Baltimore, MD, United States

**Keywords:** pectolinarigenin, breast cancer, metastasis, signal transducer and activator of transcription 3, apoptosis

## Abstract

**Background:** Breast cancer is the most common female cancer with considerable metastatic potential, which urges the need for developing novel potential drug candidate to inhibit tumor metastasis. Signal transducer and activator of transcription 3 (Stat3) have critical roles in cancer growth and metastasis and have been confirmed as a promising anticancer target. Here, we report our finding with pectolinarigenin, a flavonoid compound isolated from the aerial parts of *Cirsium chanroenicum*.

**Methods:** The role of Pec. in cell proliferation, cell apoptosis, and cell migration and invasion in three breast cancer cells (4T1, MDA-MB-231, MCF-7) was investigated. Cell proliferation was determined by MTT assay, cell apoptosis was determined by flow cytometry, and protein expression was detected by western blotting. Tumor xenograft mice model and breast tumor metastasis model *in vivo* were built to further assess the effects of Pec. on 4T1 cells.

**Results:** Intraperitoneal administrations of pectolinarigenin significantly inhibited breast cancer metastasis to lungs without affecting the tumor growth of incubated 4T1 breast cancer cells. Pectolinarigenin could also recruit CD8^+^ T cells to mediate tumor immune response. Furthermore, pectolinarigenin markedly impaired cancer cell migration and invasion by down-regulating phosphorylated-Stat3, and expression of matrix metalloproteinase (MMP)-2, MMP-9, while up-regulating the expression of TIMP2. We also found that pectolinarigenin inhibited breast cancer cell proliferation and induced apoptosis *via* mitochondrial-related apoptosis pathway, reduced mitochondrial membrane potential and the expression of Bcl-2, increased expression of Bax, and cleaved caspase-3 as well as disturbed the ROS generation.

**Conclusions:** Pectolinarigenin might potentially be a candidate for metastasis of breast cancer by mediating Stat3 pathway.

## Introduction

According to statistics, breast cancer accounts for 30% of all cancer diagnoses and ranks the second leading cause of cancer death among women (Siegel et al., 2017). It is estimated that 268,600 new cases of invasive breast cancer and 41,760 breast cancer deaths are expected to occur among US women in 2019 (Siegel et al., 2019). Similarly, in China, breast cancer alone accounts for 15% of all new cancers in women, ranking number one in 2015 (Chen et al., 2016). Despite notable improvement in survival rates of patients with breast cancer, this disease is still a major threat to women’s health, and especially patients with “triple-negative” breast cancer (TNBC), which is characterized by absent or minimal expression of estrogen receptor (ER), progesterone receptor (PR), or human epidermal growth factor receptor 2 (HER2) in tumor material (Couch et al., 2015). Advanced TNBC indicates an aggressive clinical course with a poor prognosis, comparing with non-TNBC (Dent et al., 2013). Moreover, breast cancer is highly malignant with considerable metastatic potential, and metastasis is a major problem resulting in therapy failure and lethality in patients with breast cancer (Liu et al., 2018). Poignantly, there is no effective therapy to control the recurrence and metastasis of breast cancer at present, and it is critical to develop novel therapies for breast cancer.

Currently, much effort is devoted to targeting additional genetic alterations driving breast cancer. Here, we will discuss the signal transducer and activator of transcription 3 (Stat3) signaling pathway, which has also been implicated in different types of human tumors, including melanoma, lung cancer, colorectal cancer, and breast cancer. Stat3 is a point of intersection for multiple oncogenic signaling pathways. In addition, Stat3 plays an important role in the regulation of fundamental biological processes, such as cell proliferation, differentiation, apoptosis, angiogenesis, invasion, and metastasis and inflammation (Liu et al., 2014; Zhou et al., 2017). Activated Stat3 can also down-regulate Th1 cytokines and other mediators critical for potent antitumor immune responses (Xin et al., 2011). In the case of breast cancer, the expression of phosphorylated Stat3 (p-Stat3) has been proven to be high and is associated with breast cancer progression and/or development (Alshaker et al., 2015; Yang et al., 2015). Importantly, Stat3 is constitutively activated in about 70% of breast tumors, and these breast cancers are mostly triple-negative breast tumors (Walker et al., 2014; Yang et al., 2015). What’s more, increasing evidences elucidated that blockade of Stat3 by inhibitors could trigger apoptosis and inhibit tumor growth in breast cancer (Liu et al., 2014; Ye et al., 2014; Yang et al., 2015). These researches disclosed that Stat3 inhibition provides a rational approach to the treatment of breast cancer. Although much effort has thrown into the development of Stat3 inhibitors and a number of inhibitors targeting Stat3 have been reported, no Stat3 inhibitor was approved by the Food and Drug Administration so far (Yu et al., 2013; Rath et al., 2014; Oh et al., 2015).

Traditional Chinese Medicine (TCM) is particularly valuable in disease prevention and health care. Scientific studies on TCM were last for nearly half century and most about antioxidant activity (Malviya et al., 2014; Sun et al., 2016). Pectolinarigenin (Pec.), a natural product, is mainly in the edible plants, such as *Clerodendrum volubile*, *Cirsium setidens*, *Cirsium chanroenicum*, and *Cirsium japonicum*. These plants have been used in traditional herbal medicine for a long time (Lim et al., 2008; Lu et al., 2014; Erukainure et al., 2017; Lee et al., 2017). As a flavonoid compound, Pec. could exert antioxidant, anti-inflammatory, and anticancer activities *via* increasing superoxide dismutase (SOD) activity, COX-2/5-LOX inhibition, and induction of apoptosis (Lim et al., 2008; Yoo et al., 2008; Lu et al., 2016). Pec. suppressed the tumor metastasis through Stat3 signaling inhibition in osteosarcoma (Tao et al., 2016). Considering the effects of Stat3 in breast cancer, we hypothesized that Pec., a potent inhibitor of Stat3, might be effective in the treatment of patients with breast cancer.

To test this concept, we investigated the role of Pec. in cell proliferation, cell apoptosis, and cell migration and invasion in breast cancer cells. At the same time, we constructed two models of breast cancer *in vivo* to further assess the effects of Pec. on 4T1 cells. Our results implicated that Pec. could ameliorate tumor metastasis in the lung metastasis model by inhibiting Stat3 signal pathway and increasing CD8^+^T cells. In conclusion, our results showed that Pec. may be a potential candidate in breast cancer therapy.

## Material and Methods

### Reagents and Preparation of Pectolinarigenin

All reagents, unless otherwise noted, were purchased from Sigma Chemical Co. (St Louis, MO, USA). Hoechst 33258 and the Annexin V-FITC Apoptosis Detection Kit were purchased from KeyGen Biotech (Nanjing, China). And 0.5% crystal violet was from Beyotime (Beijing, China). The primary antibodies against Stat3/p-Stat3^Tyr705^, MMP-9, cleaved caspase-3, Ki-67, Bax, and Bcl-2 were obtained from Cell Signaling Technology (Beverly, MA, USA). β-Actin and MMP-2 were purchased from ZSJQ-BIO Co. (Beijing, China) and Merck Millipore (Billerica, MA, USA), respectively. FITC-CD8a-, FITC-CD4a-, and PE-CD69-conjugated antibodies were obtained from BD Biosciences (San Diego, CA, USA).

Pec. (PubChem CID: 5320438) was purchased from Weikeqi Biological Technology Co., Ltd. (Chengdu, Sichuan, China) and has the chemical structure shown in **Supplemental Figure 1**. The purity was no less than 98% as determined by HPLC, according to the documentation from the manufacturer. For *in vitro* studies, Pec. was dissolved in DMSO at a stock concentration of 40 mM and stored at −20°C away from light. The fresh solution was prepared every 2 weeks. And it was diluted in relevant cultured medium at a final DMSO concentration of 0.1% (v/v). For *in vivo* experiments, Pec. was diluted in 5% DMSO, 35% PEG-400, and 60% physiological saline solution.

### Cell Lines and Cell Culture

Human breast cancer cell lines, MCF-7 and MDA-MB-231, as well as murine mammary carcinoma cell line 4T1 were purchased from the American Type Culture Collection (Rockville, MD, USA). The cell lines 4T1 and MDA-MB-231 in our study were authenticated using short tandem repeat analysis in March and January, 2018, respectively. Cells were maintained in DMEM or RPMI 1640 medium supplemented with 10% heat-inactived FBS (Cao Yuan Lv Ye Bio-engineering, Hohhot, China) and 1% antibiotics (penicillin and streptomycin). All cells were cultured at 37°C under a humidified 5% CO_2_ incubator.

### Cell Proliferation Assay and Colony Formation Assay

The cancer cell viability was detected using MTT assay. Dose selection referenced from relative study (Tao et al., 2016). Briefly, cells were incubated in 96-well plate overnight. On the second day, the cells were treated with various concentrations (0–40 μM) of Pec. for 24, 48, and 72 h. After that, 5 mg/ml MTT was added with 20 μl each well. Cultured for additional 3 h at 37°C, the supernatant was removed, and 150 μl DMSO was added. Finally, optical density was measured at 570 nm with a Spectra MAX M5 Microplate Spectrophotometer (Molecular Devices, CA, USA). All experiments were performed three times with five replicates.

The colony-forming ability was measured by seeding cancer cells in a six-well plate with 400–600 cells/well. The cells were treated with different concentrations of Pec. (0–40 μM) for about 12 days. At last, 0.5% crystal violet was applied to stain the colonies in absolute methanol followed cell fixed.

### Morphological Analysis by Hoechst Staining

After incubated with Pec. for 48 h in a six-well plate, cells were processed and stained with Hoechst 33258 dye according to the manufacturer’s instructions. Next, the nuclear morphology was observed by fluorescence microscopy (Olympus, BX53, Japan).

### Apoptotic Assay

MCF-7, MDA-MB-231, and 4T1 were treated with various concentrations of Pec. (0–40 μM) for 48 h. Following the manufacturer’s instruction of Annexin V-FITC Apoptosis Detection Kit, the cells were stained with FITC-conjugated Annexin V and PI. Apoptotic cells were detected by FCM (BD Biosciences).

### Mitochondrial Membrane Potential (Δψm) Assay and Detection of Reactive Oxygen Species (ROS) Level in Cells

It is generally acknowledged that Rh123 and DCFH-DA were used to test the changes of ΔΨm and ROS by FCM, respectively. In brief, cells were seeded onto a six-well plate and treated with Pec. for 48 h. Attached cells were then incubated with 1 μM Rh123 or 10 μM DCFH-DA at 37°C for half an hour in dark place. DCF, standing for ROS level, is excited by the 488 nm laser and detected at 535 nm (typically FL1-FITC). Rh123 is excited by the 488∼505 nm laser and detected at 530 nm, so we detected ΔΨm through FL1-FITC channel by FCM.

### Wound-Healing Assay

When cells grew to about 80–90% confluence in a six-well plate, they were scraped with sterile a 100 μl pipette tip, and fresh medium with 3% FBS contained different concentrations of Pec. was added. Images were taken at 0 and 48 h by an inverted microscope (Olympus, IX73, Japan).

### Boyden Chamber Migration and Invasion Assay

In brief, 1 × 10^5^ cells (for 4T1) or 5 × 10^4^ cells (for MCF-7 and MDA-MB-231) totaled in 100 μl serum-free medium were added in the upper chamber, while 600 μl medium contained 10% FBS was added at the bottom. The medium in the upper chamber and at the bottom contained various different concentrations of Pec. After 48 h, the migrated cells were fixed in methanol and then stained with 0.5% crystal violet. The images were obtained by a microscopy (Olympus, BX53, Japan). Four to six independent areas per well were counted manually. For invasion assay, 1 × 10^5^ cells (for 4T1, MCF-7, and MDA-MB-231) in 100 μl serum-free medium were plated in the upper chamber wherein the surface was coated with Matrigel. The bottom of per well in the 24-well plate was added with 600 μl medium contained 10% FBS. The cells in the upper chamber were treated with Pec. and allowed to invade for 48 h. Cells located on the underside of the filter were fixed in methanol and then stained with 0.5% crystal violet. Invasive cells were photographed by microscopy (Olympus, BX53, Japan) and counted.

### Western Blotting

Cells were treated with Pec. for 48 h. Harvest cells were lysed with RIPA buffer (Beyotime, Beijing, China) contained with protease and phosphatase inhibitor cocktail (Selleck Chemicals, Houston, USA). Protein concentrations were measured *via* the Bradford method. Protein samples run on 7.5–12% SDS-PAGE gels (Chengdu Baihe Technology Co., Ltd.) and transferred onto a polyvinylidene difluoride nitrocellulose membranes (Merck Millipore, Billerica, MA, USA). Membranes were incubated in 5% (w/v) skimmed milk buffer (skimmed milk powder/TBST) for 1 h at 37°C. After incubated overnight at 4°C, the bound primary antibodies were subsequently hybridized to corresponding secondary antibodies, and immunoreactive bands were developed with a chemiluminescence kit (Merck Millipore, Billerica, MA, USA). A monoclonal β-actin was designated as an internal reference.

### Mice, Tumor Model, and Treatment

Female Balb/c mice (6–8 weeks old) were bred and maintained in a specific-pathogen-free (SPF) condition. For the tumor xenograft mice model, Balb/c mice were injected subcutaneously with 1 × 10^6^ 4T1 cells in the right flank. Nine days after incubation, the tumor volume grew to about 200 mm^3^; tumor-bearing mice were divided randomly into three groups (n = 6 in each group) and administrated intraperitoneally injected with Pec. 25 mg/kg, 50 mg/kg, or vehicle every day. Dose selection referenced from previous study (Tao et al., 2016). Every 3 days, tumor growth was monitored by measuring length (a, mm) and width (b, mm) with a caliper, and body weight was measured. Tumor volume was calculated as a × b^2^ × 0.52. Mice were euthanized using cervical dislocation at the end of the experiment.

To build experimental breast tumor metastasis model, 2 × 10^5^ 4T1 cells in 100 μl medium without FBS or antibiotics were injected into the tail vein of Balb/c mice. After incubation for 6 days, all mice were randomly divided into two groups (n = 5 in each group). Pec. (50 mg/kg per day) or vehicle was given *via* intraperitoneal injection. Body weight of mouse was measured every 3 days. Treatments were administered for 10 days. And then all the mice were euthanized.

### Flow Cytometry

The assay was performed as previous reports (Kim et al., 2013). At the termination of tumor metastasis experiment, periphery blood and lungs were harvested. Blood and lungs single cells suspension were mechanically disrupted and enzymatically digested with 1 mg/ml collagenase IV (Gibco by life technologies, USA). Cells in periphery blood and lungs were stained FITC-CD4a-, FITC-CD8a-, and PE-CD69-conjugated antibodies for 30 min at 4°C. All samples were acquired by FCM.

### Hematoxylin and Eosin (H&E) Staining and Immunohistochemistry

In accordance with previous reports (Liu et al., 2019), tissues from mice were dehydrated, made transparent, embedded in paraffin, and then cut into sections of 3 μm thickness. These sections were stained with hematoxylin and eosin for H&E assay or primary antibodies (vimentin, Ki67, cleaved caspase-3, MMP-9, p-Stat3) using DAB Detection Kit (ZSGB-BIO Co., Beijing, China). The software ImageJ was used to statistical analyze the IHC results.

### Toxicity Evaluation

To explore the safety profile of Pec. on mice during the treatment, all the animals were observed continuously for body weight and other general conditions, including haircoat, diet, and activity. When the mice were sacrificed, the blood obtained from eyeball was used for hematological analysis. The embedded-paraffin sections of hearts, livers, spleens, lungs, and kidney were stained with H&E staining following standard protocols.

### Statistical Analysis

All data were expressed as mean ± SD of three independent experiments. The statistical significance of mean differences was determined by the unpaired two-tailed Student’s t-test with Microsoft Excel. P values less than 0.05 were designated as statistically significant.

## Results

### Pec. Blocks Spontaneous Metastasis in Mouse 4T1-Bearing Tumor Model

**Figure 1:**
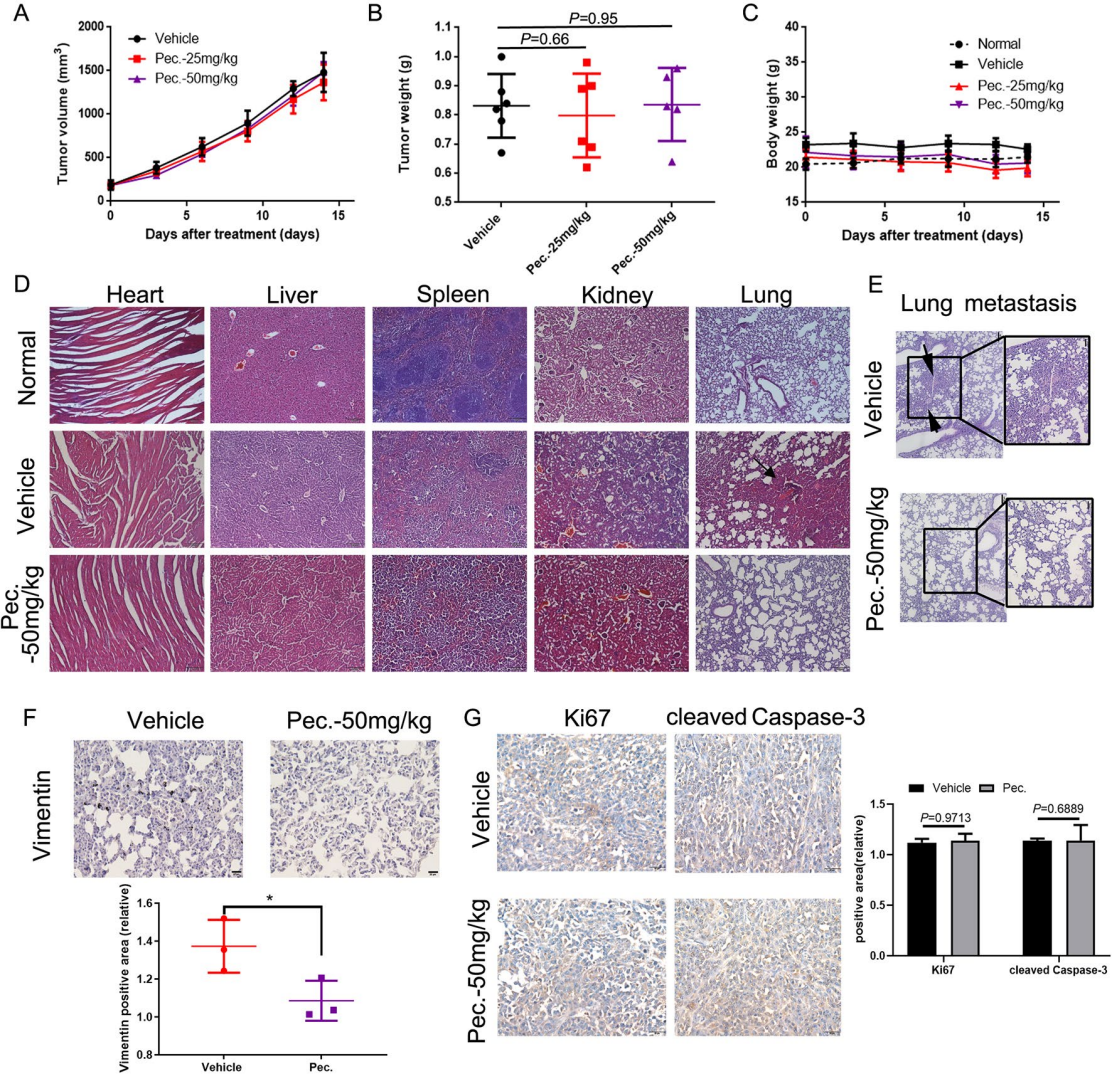
Effects of Pec. on tumor growth *in vivo*. Normal represented the untreated mice. **(A)** Tumor volumes were monitored by measuring large and short lengths with a digital caliper every 3 days and presented as mean ± SD (n = 6). No significant difference was observed between treated and vehicle group in 4T1 xenograft mice model. **(B)** Tumor weight was analyzed when the 4T1-bearing mice were sacrificed. No significant difference between treated and vehicle group in 4T1 xenograft mice model. **(C)** Mice weight was measured every 3 days. No significant difference between treated and vehicle group in 4T1 xenograft mice model. **(D)** Pec. did not cause obvious pathologic abnormalities in heart, liver, spleen, and kidney, while spontaneous metastasis nodules were appeared in the lung tissue of vehicle group in 4T1 xenograft mice model. H&E staining of paraffin-embedded sections of the heart, liver, spleen, lung, and kidney (100×). **(E)** Metastatic nodes in lung of vehicle and Pec.-treated groups using H&E staining and macroscopic photos (150×). Black arrowheads represented lung metastatic nodes. **(F)** The immunohistochemical analysis was conducted to measure the expressions of vimentin in lung tissue of 4T1 xenograft mice (400×). **(G)** The immunohistochemical analysis was conducted to measure the expressions of Ki67 and cleaved caspase-3 in primary tumor of 4T1 xenograft mice (400×). (*P<0.05 vs. vehicle control)

To explore whether Pec. could inhibit tumor growth, the 4T1 mouse breast cancer cells were injected subcutaneously into the right flank of Balb/c mice. Then 4T1-bearing mice were treated with Pec. As shown in **Figures 1A, B**, compared with the vehicle group, Pec. did not show any ability of tumor growth regression significantly. In addition, the body weight of Pec. group and vehicle group remained relatively constant (**Figure 1C**). However, 4T1 cells could spontaneously metastasize to secondary foci (lung, lymph node, and liver) from the primary sites (Zheng et al., 2018). The anti-metastatic function of Pec. could be tested *in vivo* by this model. The results of H&E staining analysis were happened to displayed that metastatic 4T1 nodules in the lungs of mice treated with Pec. (50 mg/kg) group were less than the vehicle group (**Figures 1D, E**). The augmented invasiveness was accompanied by enhanced vimentin in lung metastatic nodes, as revealed by immunohistochemistry (IHC) analyses in **Figure 1F**. No significant changes of Ki-67 and cleaved caspase-3 expressions in primary tumor were discovered between 50 mg/kg Pec. group and vehicle group (**Figure 1G**). The two results of **Figures 1A, F** were consistent. It indicated that Pec. could not inhibit 4T1 tumor growth. These interesting findings indicated that Pec. inhibited breast cancer metastasis in mouse 4T1-bearing tumor model but did not interfere with the primary tumor growth.

### Pec. Inhibits Breast Tumor Pulmonary Metastasis in Experimental Animal Model

**Figure 2:**
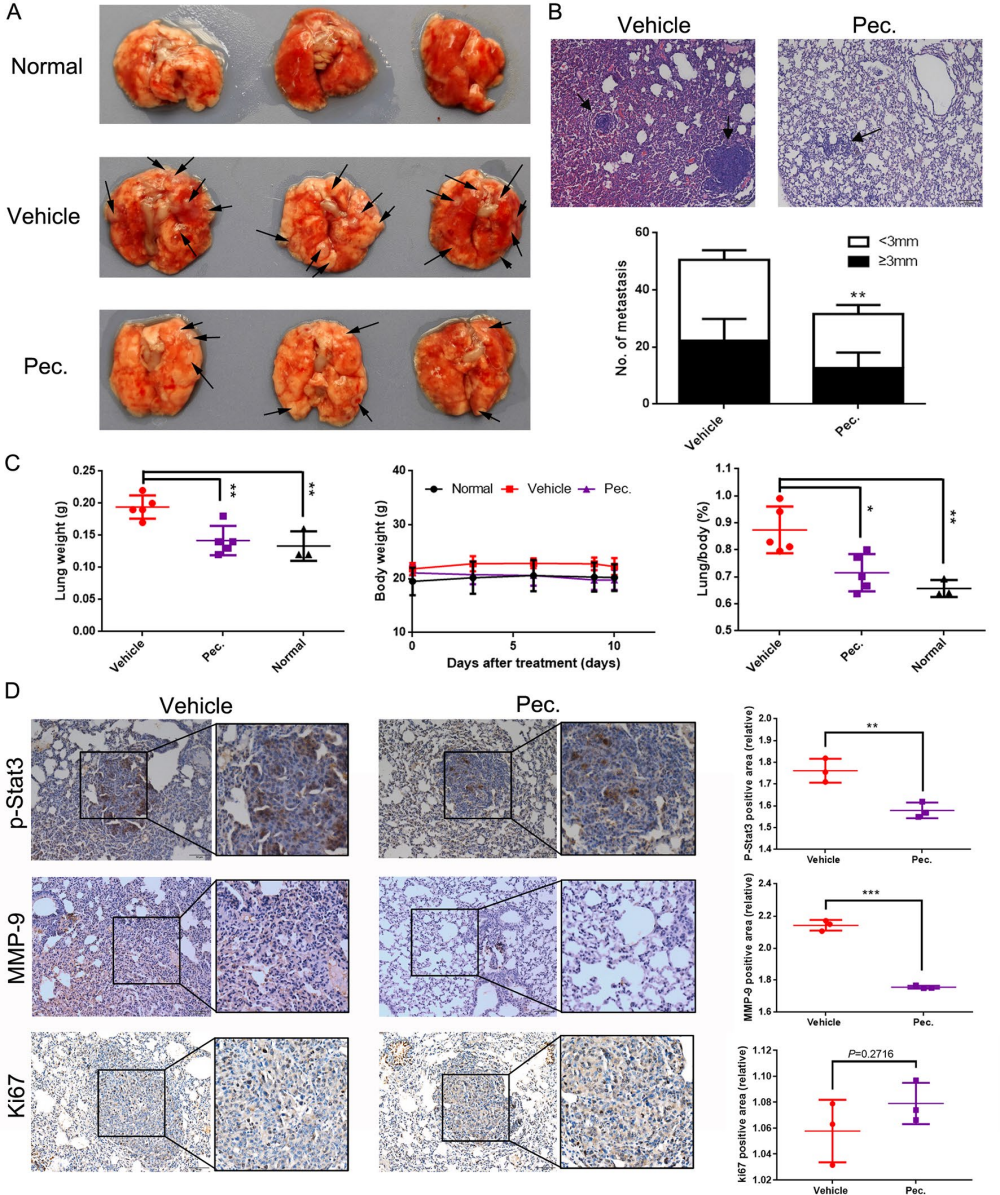
Pec. inhibited breast tumor metastasis to the lungs. 2×10^5^ 4T1 cells were injected into the tail vein to build experimental lung metastasis. Six days after injection, the mice were assigned into two groups randomly and were treated with Pec. 50 mg/kg or vehicle intraperitoneally every day. **(A)** Lung metastatic nodules were visualized. Black arrowheads represented lung metastatic nodes. **(B)** The mean lung metastasis nodules of vehicle and Pec. group at the dose of 50 mg/kg (100×). The treatment of Pec. led to significant suppression of lung metastasis. Black arrowheads represented lung metastases. **(C)** Lung weight, body weight, and the lung/body coefficients in each group. **(D)** The immunohistochemical analysis was conducted to measure the expressions of MMP-9, p-Stat3, and Ki67 in metastatic lung tissue (200×). P values for comparison of two groups were calculated by two-tailed Student’s *t*-test. (*P<0.05; **P<0.01; ***P<0.001 *vs*. vehicle control).

To further investigate the inhibitory function of Pec. on tumor metastasis, the 4T1 lung metastasis model was built by inoculating 4T1 cells intravenously into Balb/c mice. The mice received the following treatments: vehicle and Pec. at 50 mg/kg. After the mice were sacrificed, lungs were removed and weighed. Meanwhile, the lung sections were stained with H&E. In **Figures 2A, B**, a decrease in the number and size of lung metastatic nodules was seen in Pec.-treated group (**Figures 2A, B**). Moreover, as shown in **Figure 2C**, the lung weight in the treatment group significantly decreased by 30% compared with that in the vehicle group. Importantly, the lung/body coefficient decreased remarkably after treatment with Pec. compared to the vehicle group (**Figure 2C**).

We next sought mechanistic insight for anti-metastasis efficacy of Pec. through immunohistochemistry (IHC) analyses. There are increasing evidences to indicate that MMPs play pivotal roles in cancer migration and invasion (Yongxia et al., 2016). Accordingly, we determined the effects of Pec. on the expression of MMP-9 by IHC. As shown in **Figure 2D**, a lower expression of MMP-9 was observed in lung tissue after Pec. treatment. Simultaneously, we discovered that treatment with Pec. could inhibit the expression of p-Stat3 in the metastatic lung tissue (**Figure 2D**). Meanwhile, Pec. did not suppress Ki-67 expression in lung metastatic nodules. That is to say, Pec. achieved a reduction in lung metastasis nodules by inhibiting metastasis rather than inhibiting proliferation. These findings implicated that Pec. was a potential agent for inhibiting breast cancer tumor metastasis.

### Pec. Could Recruit Infiltration of T Lymphocytes in the Lung Metastasis Model

**Figure 3:**
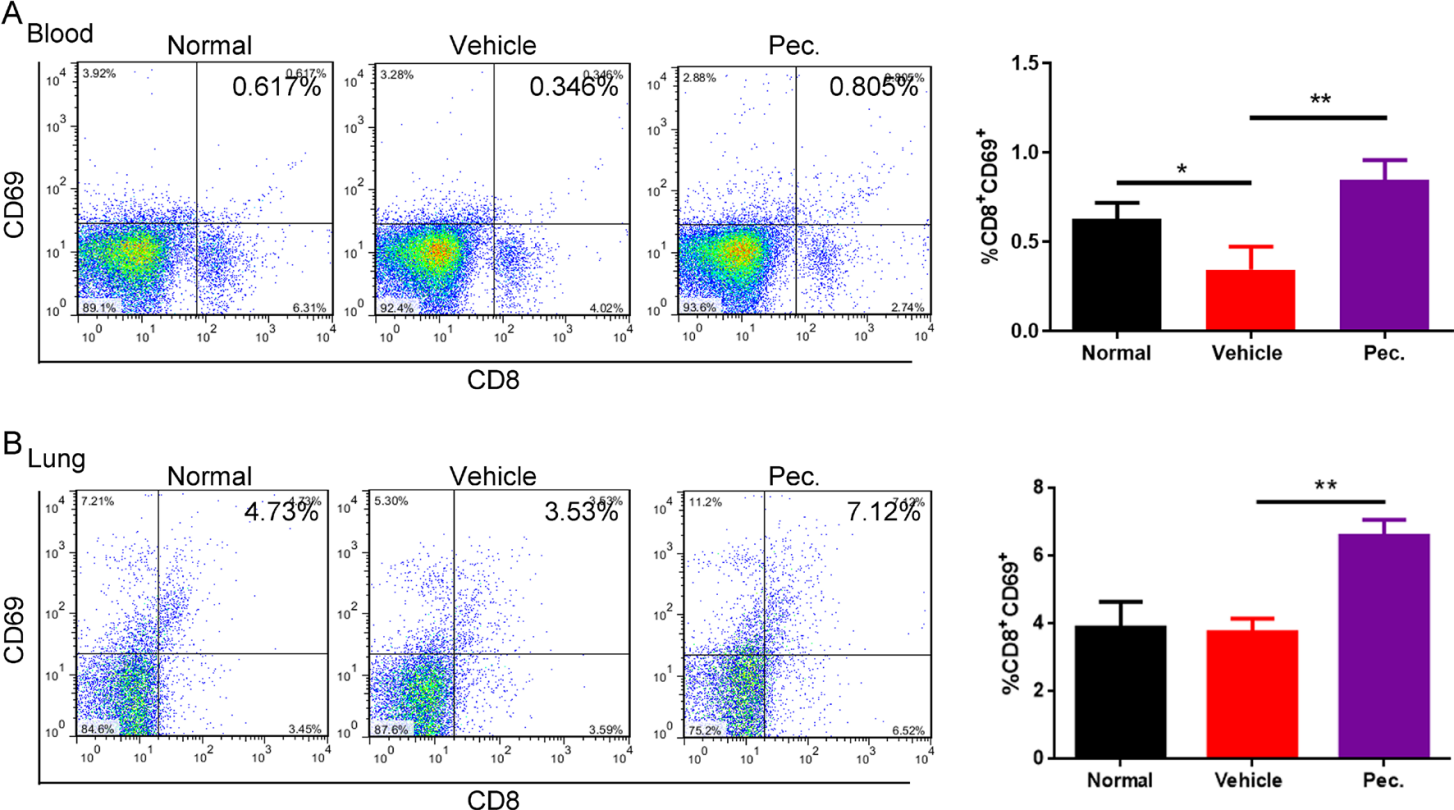
Pec. recruited CD8^+^T cells in periphery blood and lung tissues. FCM was used to determine the expression of CD8^+^T cells. These cells were isolated from blood **(A)** and lungs **(B)** of experimental 4T1 lung metastasis model mice treated with vehicle, or Pec. at 50 mg/kg and normal mice as negative contrast. Statistic results were presented as mean ± SD (n = 3, *P<0.05, **P<0.01).

The immune system could serve as an extrinsic tumor suppressor. T lymphocytes are recognized as immune mediator response against to cancer, and lack of tumor-specific CD8^+^T cells could promote tumor immune evasion (Ting-Hong et al., 2017). Hence, we measured active CD8^+^T cells in peripheral blood and metastatic lung tissue of lung metastasis mice model. Active CD8^+^T cells were characterized by CD8^+^ and CD69^+^ double-positive cells by FCM. As shown in **Figure 3**, the active CD8^+^ T cells in peripheral blood and metastatic lung tissue were both increased in the Pec.-treated group compared with the vehicle control. These data suggested that Pec. might be also mediate specific anti-tumor immunity and efficiency through inducing the production and accumulation of CD8^+^T-cells *in vivo*.

### Pec. Suppresses Breast Tumor Cell Migration and Invasion *In Vitro*

**Figure 4:**
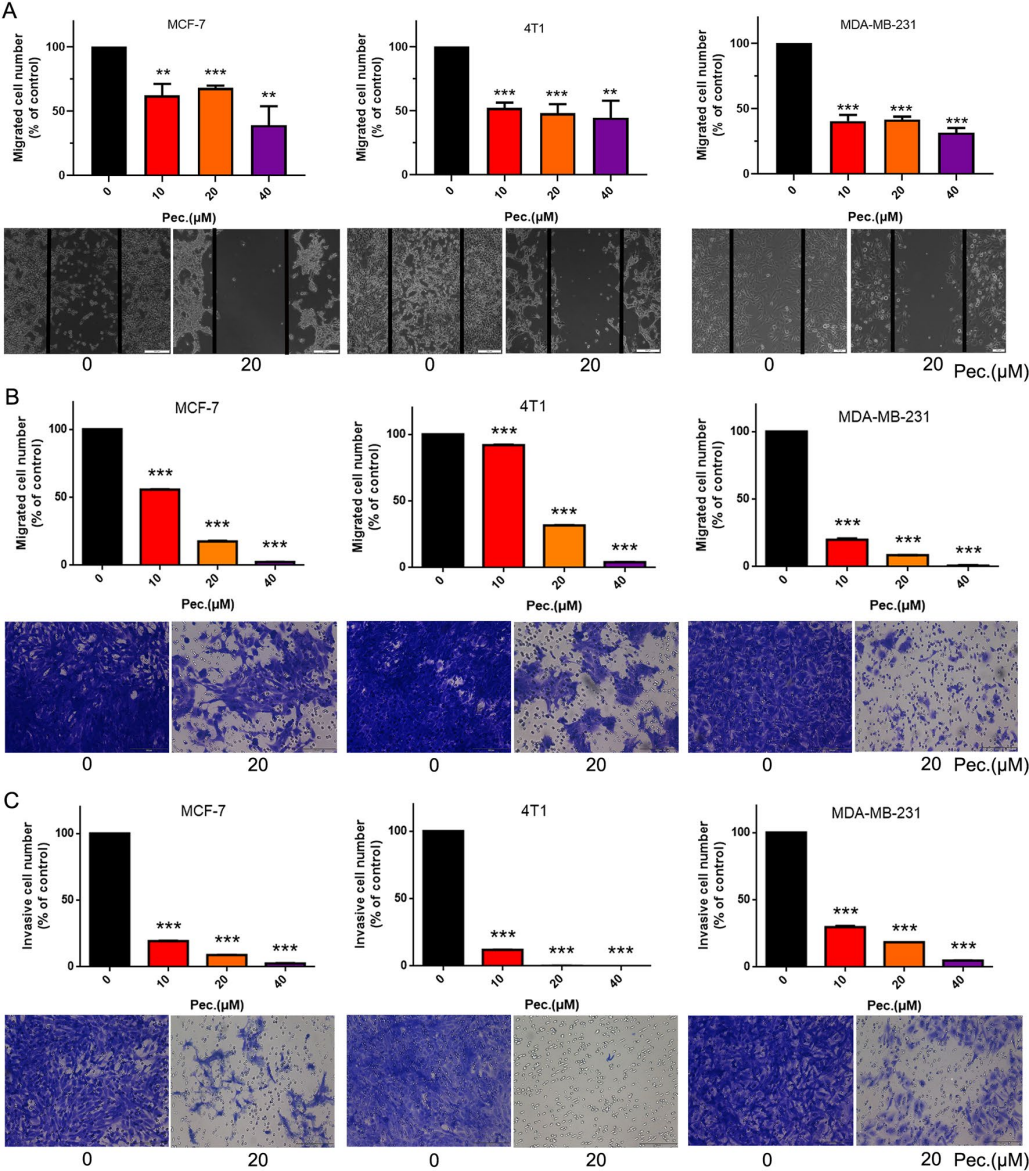
Pec. suppressed breast cancer cells MCF-7, 4T1, and MDA-MB-231 migration and invasion. **(A)** Pec. inhibited theses three breast cancer cells, MCF-7, 4T1, and MDA-MB-231, migration in wound healing assay (100×). In the wound healing assay, cells were seeded into the six-well plate. A wound was created when the cells grew into 80–90% confluence. After incubation for 48 h the groups were fixed and imaged. The lines represent the area occupied by the initial scraping, and migrated cells were counted. **(B)** MCF-7, 4T1, and MDA-MB-231 cells were implanted in the top chamber of transwell with serum-free medium and treated with Pec. (200×). The cells were fixed, stained, and quantified after 48 h. **(C)** Pec. restrained the invasion of breast cancer in transwell assay (200×). Appropriate number of cells were seeded on the upper chamber membrane where were pre-treated with Matrigel and treated various concentrations of Pec., while the bottom chamber was filled with medium contained 10% FBS. Results are representative from three parallel experiments. (**P<0.01; ***P<0.001 compared to vehicle control).

Abovementioned *in vivo* results indicated that Pec. could inhibit breast carcinoma metastasis, and we queried the effect of Pec. on breast tumor cells *in vitro*. Wound healing and transwell assays on MCF-7, 4T1, and MDA-MB-231 were conducted to further explore the effects of Pec. on migration and invasion *in vitro*. As shown in **Figure 4A**, the wound healing assay implied that Pec. significantly inhibited the migration in three breast cancer cell lines while the cells were found to migrate to the wound area in the control group. Similar results were obtained in migration assays in **Figure 4B**. In addition, invasion assays assessed the ability of 4T1 cells to invade through the Matrigel; 20 μM Pec. almost inhibited 4T1 cell invasion (**Figure 4C**). Similar results were observed for MCF-7 and MDA-MB-231 cells.

**Figure 5:**
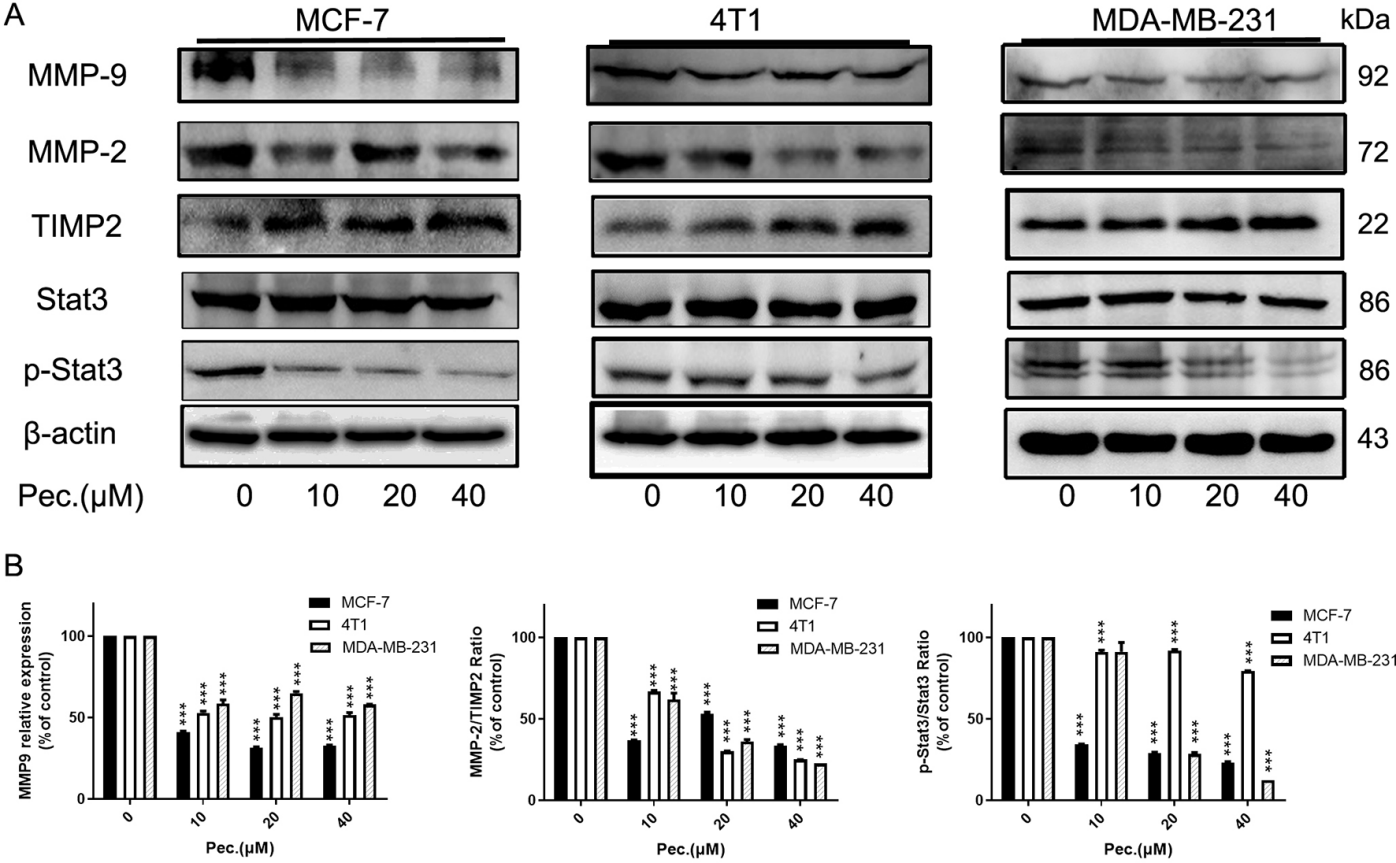
Pec. down-regulated the expressions of invasion related proteins. **(A)** Western blot analysis of MCF-7, 4T1, and MDA-MB-231 following Pec. treatment, including the expressions of MMP-9, MMP-2, TIMP-2, and Stat3/p-Stat3. β-actin served as loading control. **(B)** The relative expressions were quantified with Image-Pro Plus. (***P<0.001 *versus* vehicle control).

For further verify anti-migrate and invasive effects of Pec., some protein expressions were detected. MMP-9, p-Stat3, MMP-2, and TIMP-2 are considered to be involved in cell migration and invasion (Tsui et al., 2018). The results of western blot suggested that Pec. could significantly inhibit the expressions of MMP2, MMP-9, and p-Stat3, while up-regulating the expression of TIMP-2 without affecting the total Stat3 expression level in MCF-7, 4T1, and MDA-MB-231 cells (**Figures 5A, B**). Taken together, these results implied that Pec. inhibited the migration and invasion of breast cancer cells though STAT3/MMP pathway.

### Pec. Diminishes Breast Cancer Cell Proliferation and Triggers Cell Apoptosis

**Figure 6:**
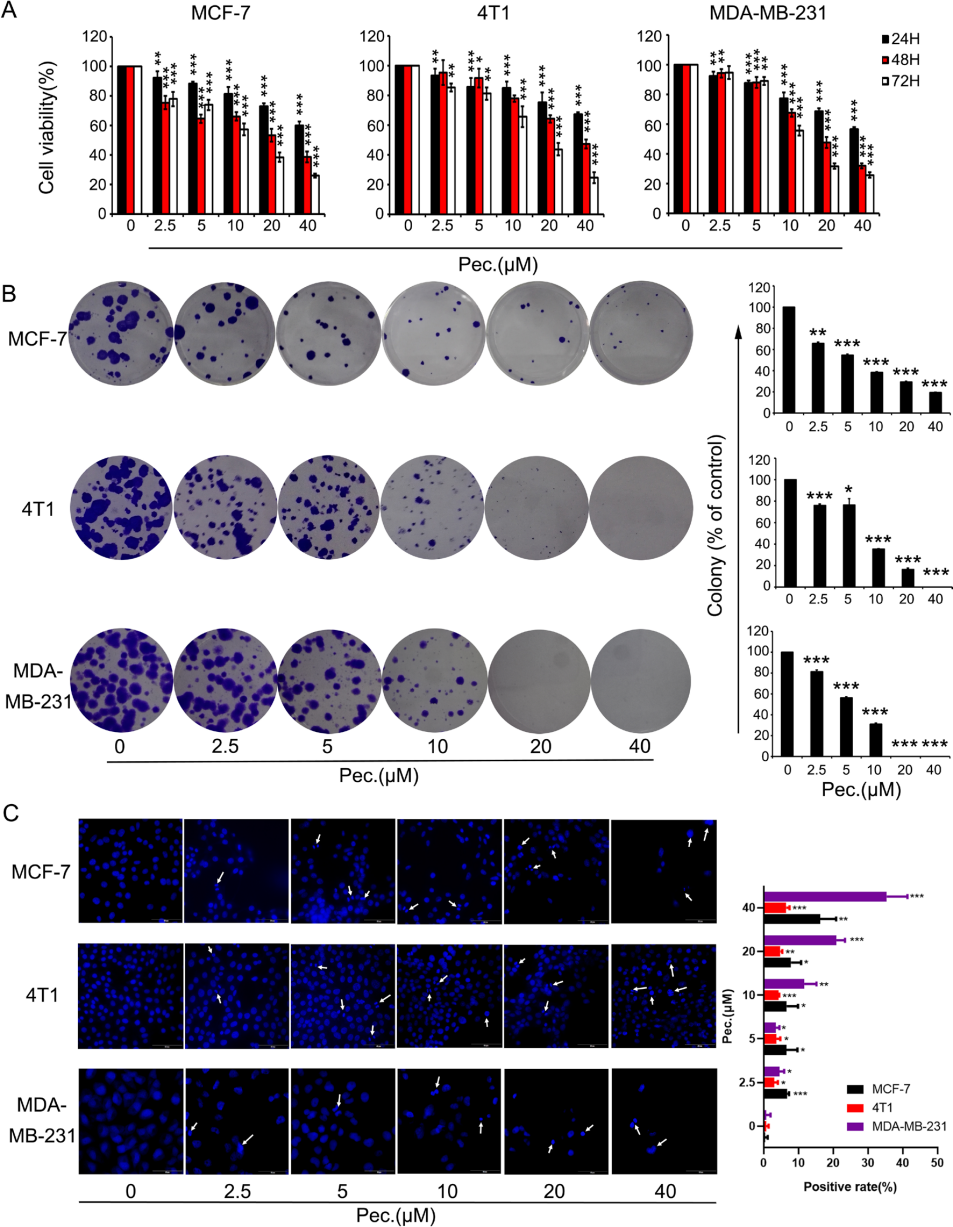
The effects of Pec. on viability in breast cancer cells. **(A)** Breast cancer cells MCF-7, 4T1, and MDA-MB-231 were treated with different concentrations of Pec. for 24, 48, or 72 h, and cell viability was detected *via* MTT assay. Each bar represents the mean ± SD for three independent experiments. **(B)** The effects of Pec. on colony formation in these three breast cancer cell lines. After incubation for about 12 days, the colonies were fixed, stained, and counted manually. Data are expressed as mean ± SD from three experiments. **(C)** The fluorescence microscopic appearance of Hoechst 33258 staining nuclei of MCF-7, 4T1, and MDA-MB-231 cells with various concentrations of Pec. for 48 h (400×). The apoptotic nuclear was defined as positive. The positive rate was quantitated. Results are representative from three parallel experiments. (*P<0.05; **P<0.01; ***P<0.001 *vs*. vehicle control).

To determine whether Pec. has direct effects on breast tumor cells, we tested its ability to kill MCF-7, MDA-MB-231, and 4T1. In this case, MTT assay and clonal formation assay were performed. As shown in **Figure 6A**, Pec. could suppress breast cancer cell viability in a time- and concentration-dependent manner. The IC_50_ values of Pec. were provided in **Supplementary Table 1**. The 48 h IC_50_ values of these three cell lines were between 23 and 40 μM. Moreover, the clone formation of MCF-7, 4T1, and MDA-MB-231 cells were lessened in a dose-manner after exposure to Pec. for 12 days or so. Notably, the size of colonies handled by Pec. was smaller than the control groups in these three cell lines (**Figure 6B**). Above analyses suggested that Pec. have a strong cytostatic and cytotoxic effects on breast cancer cells. The anti-proliferative effect of Pec. on MDA-MB-231 is better than the other two cell lines.

When cells execute apoptosis, resulted from nuclear condensation and fragmentation, the fluorescence intensity of nucleus stained with Hoechst 33258 was stronger than that in normal cells. The **Figure 6C** implied that Pec. induced apoptosis after 48 h treatment, along with the reduction of cell number. In addition, these changes adhered to a dose-dependent manner. Annexin V-FITC/PI kit was employed to test the presence of apoptotic cells. As shown in **Supplementary Figures 3A, B**, after Pec. treatment for 48 h, the apoptosis induction effects in MCF-7, 4T1, and MDA-MB-231 cells were discovered. When MDA-MB-231 cells were treated with 40 μM Pec. for 48 h, the apoptosis rate was increased remarkably from 2.69 to 42.6%, whereas the percentage of MCF-7 apoptosis cells was increased from 6.31 to 28.6%. It was elucidated that Pec. was able to induce apoptosis in a concentration-dependent manner. Nevertheless, the change of 4T1 apoptosis rate was less significant, which may partly explain why Pec. could not inhibit the tumor growth in 4T1-bearing mice. Taken together, the suppression of breast cancer cells by Pec. may be mediated by the induction of apoptosis in part.

### Pec. Induced Apoptosis *via the* Mitochondria-Mediated Apoptotic Pathway

In order to further identify that Pec. induced cell death was related to apoptosis, we measured the levels of Bcl-2, Bax, and cleaved caspase-3 in MCF-7, 4T1 and MDA-MB-231 cells after Pec. treated for 48 h. As shown in **Supplementary Figures 3C–E**, the expressions of Bcl-2 in these three cell lines decreased, while that of cleaved caspase-3 in three breast cancer cell lines significantly increased after Pec.-treated. These variations of protein levels indicated that the mitochondrial apoptotic pathway might be involved in Pec.-induced apoptosis in breast cancer cells.

The changes in the mitochondrial membrane potential (ΔΨm) were detected to confirm the hypothesis. Stained with Rh123 and measured by FCM, ΔΨm was reduced obviously, especially in 4T1 and MDA-MB-231 cells (**Supplementary Figures 4A, C**). Aimed to further verify the mechanism, reactive oxygen species (ROS) generation was tested by FCM stained with fluorescent dye, DCFH-DA. As shown in **Supplementary Figures 4B, D**, the increased levels of ROS were occurred when MCF-7 and 4T1 cells were treated with Pec. for 48 h. Nevertheless, the decreased level of intracellular ROS was observed in MDA-MB-231 cells. No matter how the ROS generation changes, it always has influence on the intracellular environment, which may make further efforts to trigger cell apoptosis. Generally, these data warranted a better understanding of molecular mechanism that underline Pec.-induced apoptosis in breast cancer cells.

### Toxicity Evaluation

As mentioned above, during the treatment of Pec. on 4T1 tumor-bearing mice and lung metastasis mice, no obvious adverse effects were observed in Pec.-treated groups, such as toxic death, body weight loss, and skin ulceration. As observed by microscopy, compared with vehicle group, no pathologic changes after Pec. treatment were found in the heart, liver, spleen, and kidney (**Figure 1D**). Additionally, in the lung metastasis model mice, serological and hematological analyses did not show any pathological changes. No significant differences in body weights were observed between Pec. group and normal group. Only TP (serum total protein) of vehicle group was slightly higher than that of Pec.-treated group (**Figure 2C** and **Supplementary Figure 2**).

## Discussion

As the most common cancer among females, breast cancer is the primary cause of cancer mortality with considerable metastatic potential. Tumor metastasis accounts for the most deaths from breast cancer (Zhang et al., 2012). Although advance in the treatment for metastatic breast carcinoma have increased the survival rate, metastatic breast cancer is not curable yet (Luu et al., 2011). Accordingly, it’s necessary to unearth the novel potential drug candidate against breast tumor. Moreover, recent studies have confirmed that Stat3 is activated in breast cancer, and its overexpression was closely related to the development of breast cancer (Chang et al., 2013; Egusquiaguirre et al., 2018). Interestingly, LIF receptor/STAT3 signaling is proved to be a dormancy phenotype to disseminated breast cancer cells, and inactivation of JAK/STAT pathway might contribute to disease progression and metastasis (Johnson et al., 2016). However, the vital discovery is verified in strongly hypoxic sites, such as the bone marrow, and it is uncertain that the same mechanism will work in the same way in other metastatic sites, such as the lung. Therefore, targeting Stat3 might be still an attractive approach for breast cancer and lung metastasis therapy.

The current study was performed to investigate the effect of Pec., a natural product, on breast cancer metastasis and provide insight into its underlying mechanism. Recent study has indicated that Pec. may be a novel approach for osteosarcoma intervention because of its Stat3 signaling inhibitory activity (Tao et al., 2016). The important findings of our study were that Pec. impaired breast cancer metastasis *in vivo* blocked cancer cells migration and invasion, as well as induced apoptosis *in vitro*. In addition, Pec. exerted anti-breast cancer metastasis activities *via* impairing the Stat3 signaling pathway in part while improved apoptosis in breast cancer cells by mitochondrial-related pathway.

Based on our intention to mechanistically dissect the role of Pec. in tumor metastasis, we first assessed the effects of Pec. on tumor metastasis *in vivo*. Pec. was shown to significantly restrain mouse breast cancer metastasis to the lungs in the subcutaneous 4T1-bearing mice models. Nevertheless, the growth of subcutaneous tumor was not suppressed by Pec. Besides, administration of Pec. triggered the decreasing of tumor metastasis nodes remarkably in 4T1 lung metastasis model. IHC assay results indicated that Pec. inhibited the expressions of p-Stat3 and MMP-9, which are generally acknowledged to be correlated positively with tumor metastasis (Yang et al., 2015; Thakur et al., 2018). The reliably researches have proven that the infiltration of CD8^+^ T cells could reduce the relative risk of death from breast cancer (Mahmoud et al., 2011; Ali et al., 2014). Additionally, it is well known that CD8^+^ T cells response could be inhibited by tumor-associated macrophages (TAMs), which are participated in immune suppression and tolerance in the cancer microenvironment (Saio et al., 2001; Condeelis and Pollard, 2006). Our data implicated that Pec. could recruit CD8^+^ T cells in periphery blood and lungs compared with that of vehicle group in lung metastasis model. It is therefore conceivable that Pec. could restrain breast cancer metastasis by suppressing Stat3 pathway and recruitment of CD8^+^ T cells.

Cancer cell migration and invasion are the critical steps in successful cancer metastasis, and suppression these steps is a vital approach to antitumor treatment (Peter and Katarina, 2003). Thus, wound healing, transwell migration, and invasion assays were conducted to elucidate the anti-metastasis ability of Pec. The invasive tumor cells need to degrade the extracellular matrix through metalloproteinase (MMPs) and then alter cell-to-cell junction and cell adhesion. Stat3/MMP-involved pathway is essential for cancer invasion and metastasis (Ting-Hong et al., 2017). Pec. was found to inhibit Stat3 phosphorylation activity and decrease the expression of proteins, including MMP-9 and MMP-2. Similar results were observed in the lung metastasis model, with reduction of MMP-9- and p-Stat3-positive metastasis areas by IHC analysis. On the other hand, TIMP2 suppresses their activity and is therefore deemed to own inhibitory effect on cancer metastasis (Curran and Murray, 1999). Pec. notably up-regulate the expression of TIMP2 in MCF-7, 4T1, and MDA-MB-231 cells. These results further validate the efficacy of Stat3/MMP signal pathway blockade by Pec. for anti-metastasis function on breast carcinoma.

ROS plays as powerful molecules to active or inactive signaling pathway involved in a variety of biological processes. As reported, accumulation of ROS results in activation of STAT3 signal pathway as well as overexpression of MMP2 and MMP9 (Yang et al., 2013). In breast cancer, oxidative stress promotes metastasis through inducing angiogenesis, p38 MAPK and MMPs (Brown et al., 2000; Rajagopalan, 1996; Wang et al., 1998). In our study, Pec. decreased ROS of MDA-MB-231 and phosphorylation of Stat3, inhibiting cell metastasis. However, in MCF-7 and 4T1 cell lines, Pec. increased the accumulations of ROS. These differences may result from the diversity of cell line, TNBC and non-TNBC. The effects of PEC on ROS are different in TNBC and non-TNBC cells, and the role of ROS in metastasis is further affected. On the other hand, STAT3 may be one of the targets of Pec. in MCF-7 and 4T1 cells. The mechanisms of PEC against breast cancer metastasis need further exploration, and whether the mechanism is associated with ROS.

Of note, we also conducted assays to identify the functions of Pec. on breast cancer cells. The MTT results indicated that Pec. inhibited the proliferation of breast cells in a time- and dose-dependent manner, and colony formation outcomes were consistent with this result. Moreover, a diverse range of cell signal pathways participated in apoptosis, which is a major route to eradicate tumor cells (Elmore, 2007). In the intrinsic apoptosis pathway, the mitochondria exert a pivotal role by changing the mitochondria transmembrane potential. Bcl-2 family proteins, embracing the antiapoptotic protein Bcl-2 and the proapoptotic protein Bax, take part in this apoptosis pathway (Wang and Youle, 2009). In this study, Hoechst 33258 staining and FCM assays both indicated that Pec. could trigger the apoptosis of breast cancer cells. Additionally, the activation of Bax and cleaved caspase-3 as well as downregulation of Bcl-2 were observed. The treatment of Pec. also induced the loss of ΔΨm. Release of proapoptotic factors from mitochondria into the cytoplasm further cause apoptosis events. These results demonstrated that Pec. could suppressed breast cancer growth through apoptosis induction and proliferation inhibition, which is consistent with previous studies (Lu et al., 2014; Lu et al., 2016). Moreover, the abnormality of the ROS homeostasis has been involved in mitochondrial dysfunction and apoptosis (Kim et al., 2013; Zheng et al., 2013). The results suggested that Pec. could significantly increase ROS production in MCF-7 and 4T1 cells while decreased slightly that in MDA-MB-231. In other words, Pec. could break the homeostasis of the intracellular environment. Therefore, further studies are still urged to explain this phenomenon. Taken together, mitochondrial-related apoptosis pathway was involved in Pec.-induced apoptosis among breast cancer cells. However, it was reported that Pec. could inhibit osteosarcoma growth and metastasis and reduce breast cancer cell (MCF-7) tumorigenicity *in vivo* (Lu et al., 2016; Tao et al., 2016). The advantage and disadvantage inactivations of STAT3 are quite different in the course of disease occurrence and development, which are also influenced with microenvironment (Johnson et al., 2016; Segatto et al., 2018). No matter in 4T1 spontaneous tumor or metastatic model, Pec. did not exert anti-proliferation effect. In this respect, the results *in vitro* and *in vivo* do not match. We assume that the low inhibitory rate and lung metastasis *in vivo* might be due to the low dose of Pec., its poor bioavailability, and microenvironment conditions. It calls for further study. Accordingly, deeper researches should be conducted to explain and strengthen its antitumor activity *in vivo*. Moreover, from the results *in vitro*, Pec. may exert stronger antitumor and anti-metastasis activities on MDA-MB-231 cell lines. This cell line is a kind of human TNBC cell lines. It indicated that Pec. may have significant effect on TNBC. The TNBC cells lack the necessary receptors; common treatments like hormone therapy and drugs that target estrogen, progesterone, and HER-2 are ineffective. Using chemotherapy to treat triple negative breast cancer is still an effective option. Exploring the underlaying mechanism of Pec. on TNBC is more valuable, which could be our future research direction. In conclusion, above results presented here are to our knowledge the first evidence that Pec. is a potential agent in inhibiting breast cancer metastasis. Mechanism researches demonstrated that Pec. could restrain breast cancer metastasis by blockage Stat3/MMP pathway *in vivo* and *in vitro*. Besides, Pec. could recruit CD8^+^ T cells in lungs and blood in lung metastasis mice model. In addition, our present work also implied that Pec. could suppress cancer cell growth and induce apoptosis *via* mitochondrial-related apoptotic pathway *in vitro*. Thus, we believe that Pec. may be a promising agent in breast cancer chemotherapy by targeting Stat3 signaling pathway.

## Data Availability Statement

The datasets generated for this study are available on request to the corresponding author.

## Ethics Statement

All mouse experiments were approved and performed in compliance with the Animal Care and Use Committee of Sichuan University in China (New Permit Number: 20160220).

## Author Contributions

YL, WY and TY designed the experiments. YL, CG, YaZ, CF, LW and YY performed the experiments. YL, YD, QZ and TY collected and analyzed the data. YiZ, FH and YX provided reagents and advice. YL, XY, WY and TY wrote and edited the manuscript.

## Funding

This work was supported by Key Project of the Science & Technology Department of Sichuan Province (Grant No. 2017JY0071); Graduate Student’s Research and Innovation Fund of Sichuan University (2018YJSY110).

## Conflict of Interest

The authors declare that the research was conducted in the absence of any commercial or financial relationships that could be construed as a potential conflict of interest.
