# Supplementary material for: Inhibition of Stat3 Signaling Pathway by Natural Product Pectolinarigenin Attenuates Breast Cancer Metastasis
Source: Front Pharmacol. 2019 Oct 10;10:1195. doi: 10.3389/fphar.2019.01195 (PMC6796319; doi:10.3389/fphar.2019.01195)

Supplementary Material

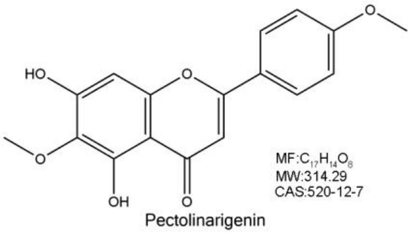

Supplementary Figure 1. The chemical structure of Pec..

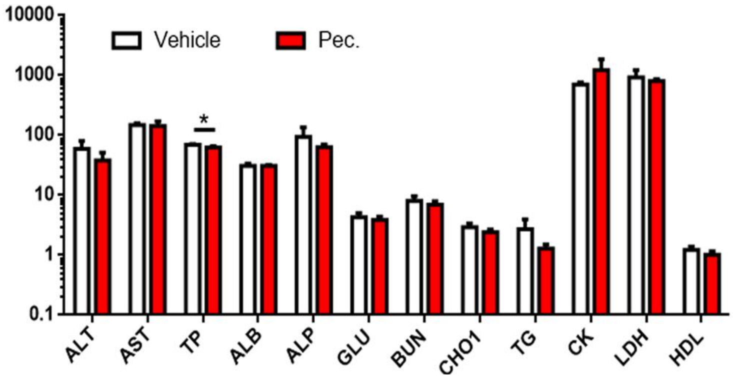

Supplementary Figure 2. Effects of Pec. on serum biochemistry of blood in 4T1 lung metastasis mice model. Serum biochemistry analysis of blood was done when the mice were euthanized. Units of the parameters are as follows: ALT, AST, ALP, CK, LDH, U/L; TP (total protein), ALB, g/dl; GLU, BUN, HDL, mg/dl; CHO1, TG, mM. (*P＜0.05 compared to vehicle group)

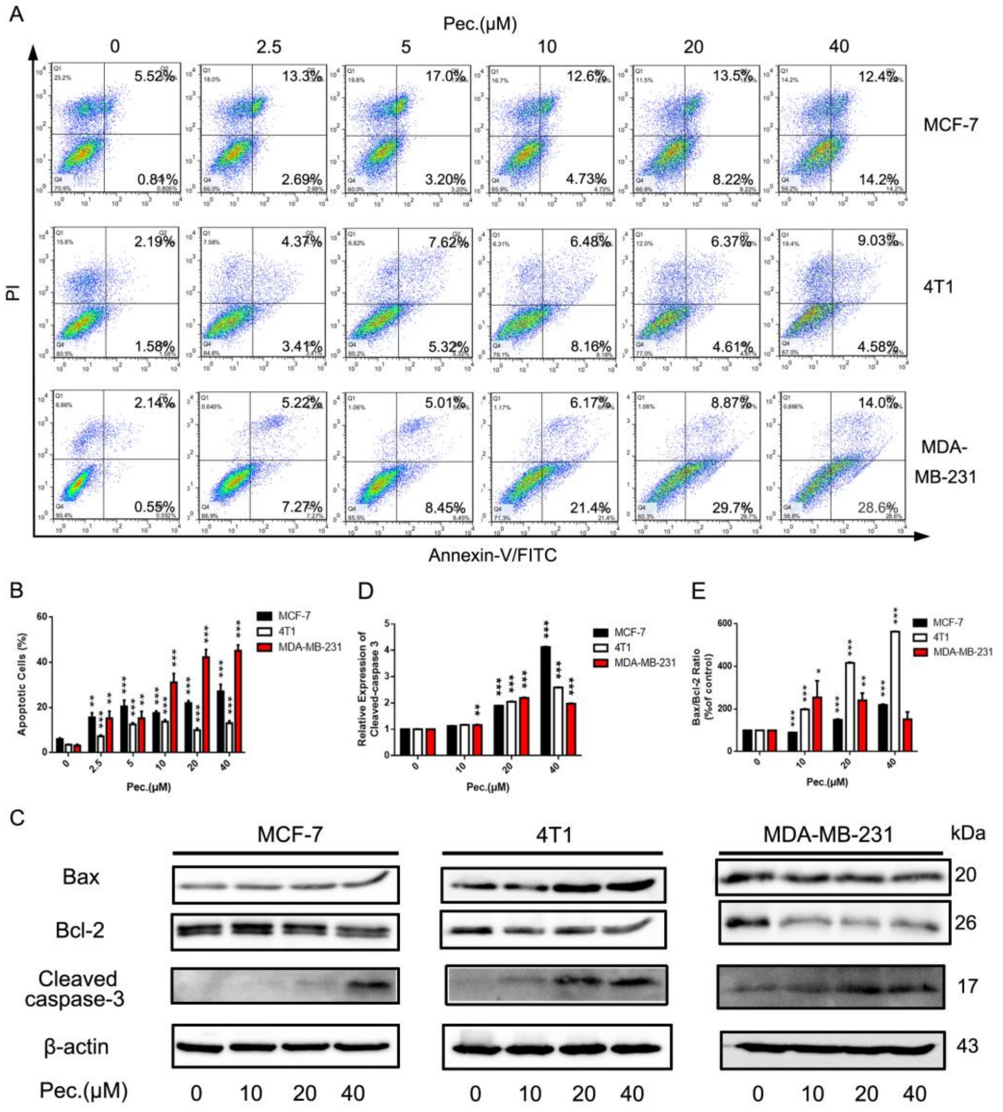

Supplementary Figure 3. Induction of apoptosis of MCF-7, 4T1 and MDA-MB-231 cells by Pec. treatment. (A) These three cell lines were treated with Pec. at indicated doses for 48h. The Annexin V-FITC/PI kit was used to test the apoptotic cells. (B) The apoptosis rate was treated statistically. Data are expressed as mean±SD. (C, D, E) Western blot assay of three breast cancer cell lines treated with Pec. to evaluate protein expression of Bax, Bcl-2, Cleaved caspase-3 and β-actin was used as a standard. The relative expressions of Cleaved caspase-3 and Bax/Bcl-2 ratio were quantified with Image pro plus. (**P＜0.01; ***P＜0.001 compared to vehicle control)

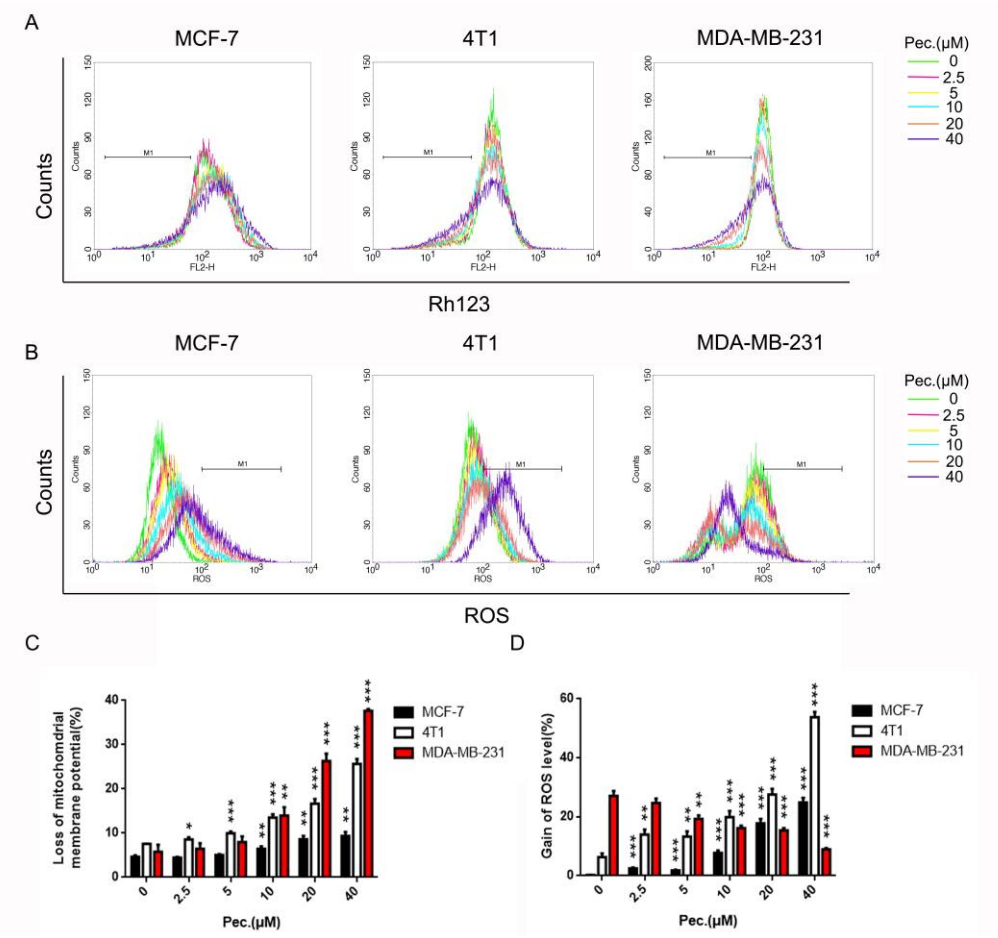

Supplementary Figure 4. Impact of Pec. on intrinsic apoptosis pathway. (**A**, **C**) Pec. decreased the mitochondrial membrane potential (ΔΨm) in MCF-7, 4T1 and MDA-MB-231 cells. Cells were treated for different concentrations of Pec. for 48h and then stained by Rh123 to detect the changes of ΔΨm by FCM. The bars represent the loss of $\triangle\Psi_m$ and are shown as mean±SD. (**B**, **D**) Pec. increased the levels of ROS in MCF-7 and 4T1, while decreased that in MDA-MB-231. These three cell lines were treated with Pec. for 48h. The harvested cells were stained with DCFH-DA and then measured by FCM. Quantification of ROS is also shown. (*P＜0.05; **P＜0.01; ***P＜0.001 vs vehicle control)

Supplementary Table 1. The IC50 values of three breast cancer lines in MTT assay (n=3).

Cell line	MCF-7	4T1	MDA-MB-231
48h	30.6±13.2μM	39.8±0.6μM	23.1±5.5μM
72h	18.7±8.7μM	15.7±0.6μM	14.9±1.8μM

MTT assay was employed to detect the cell viability. Each cell line was treated with various concentrations of Pec.. Data are expressed as the mean from three experiments.

Wound healing Assay

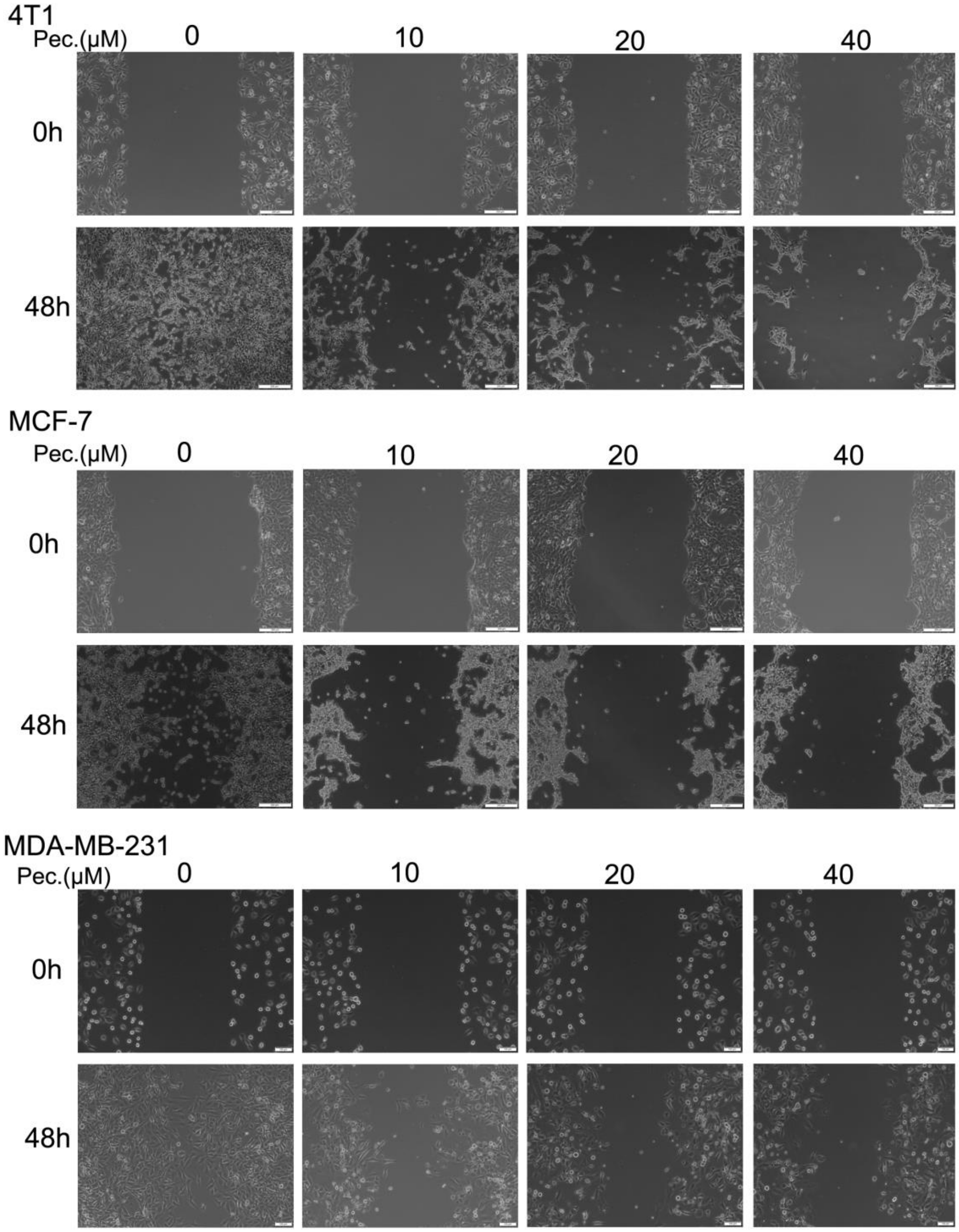

Figures of Full Length Western Blots

1.Western blot in Figure 5A

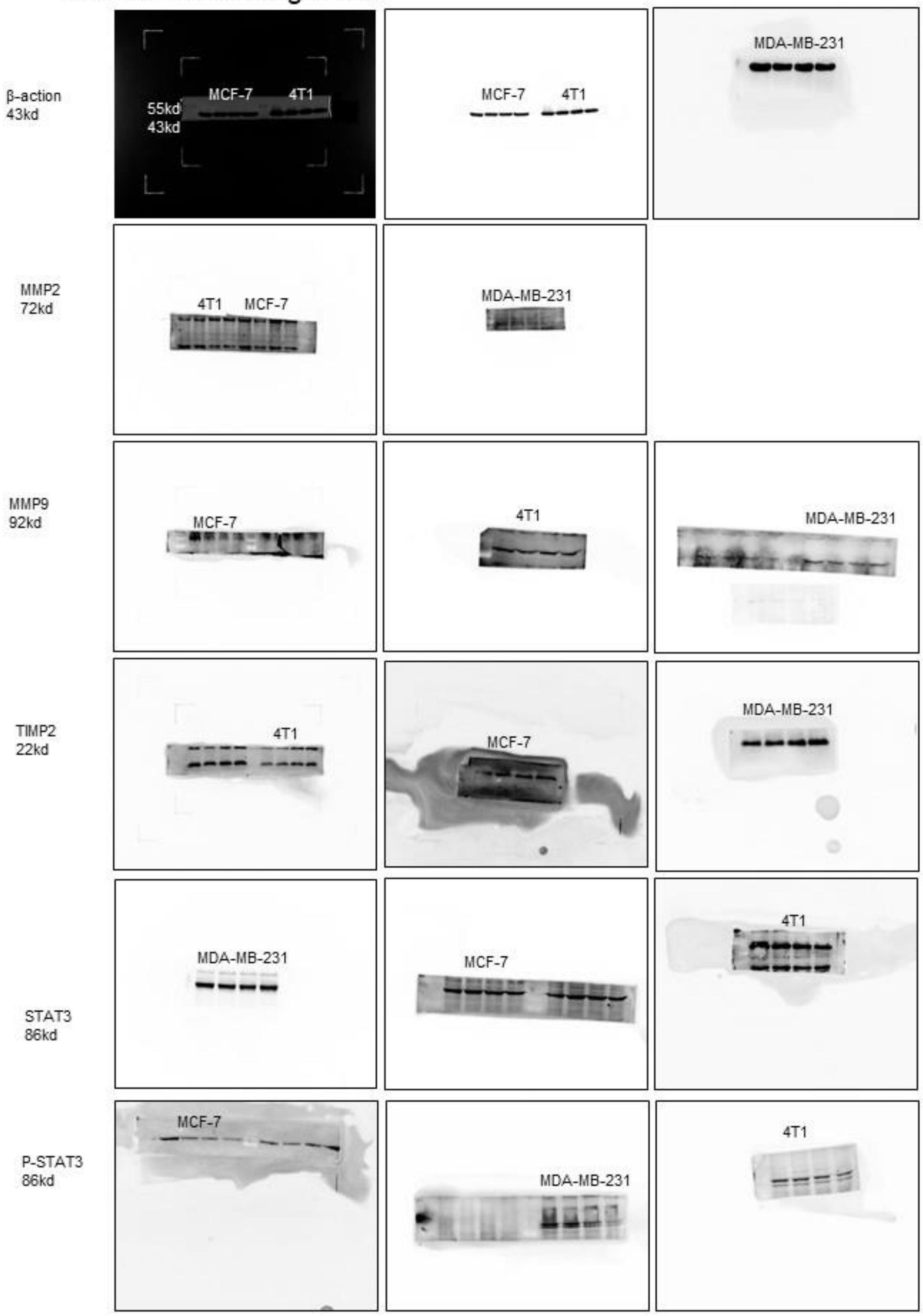

2. Western blots in Supplementary Figure 3C

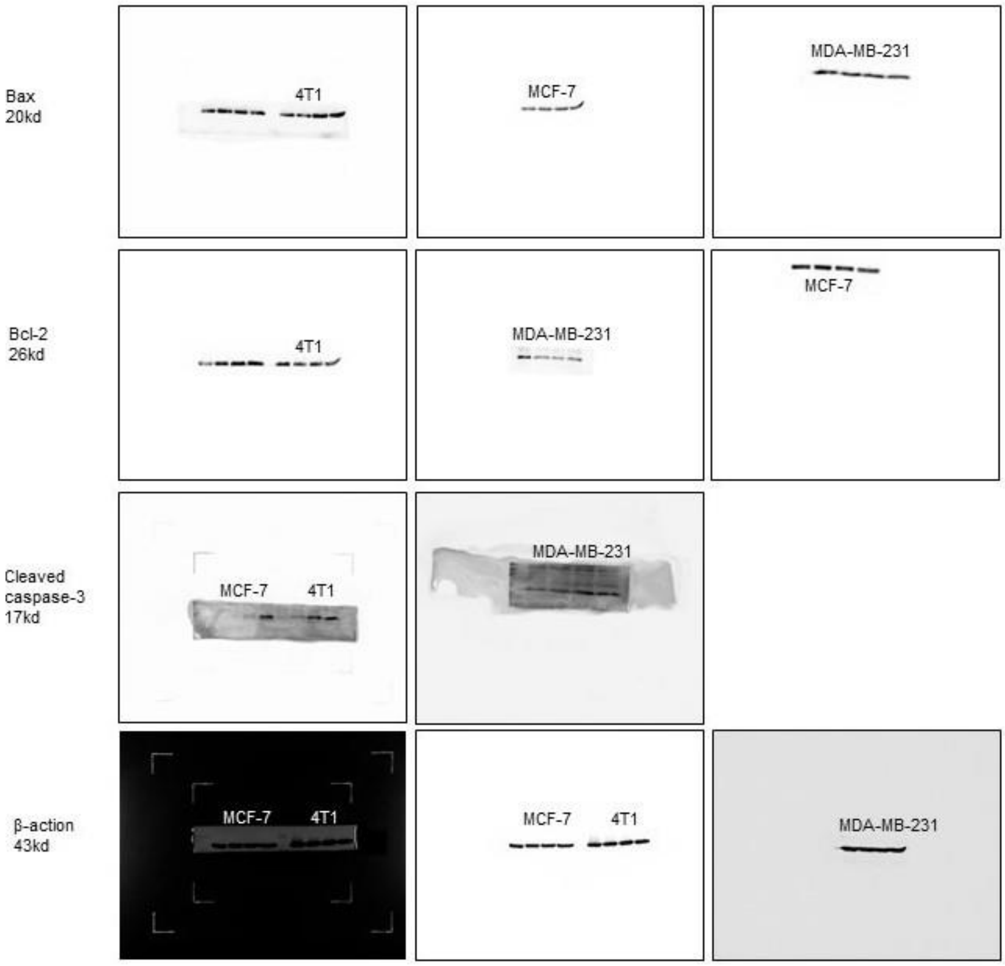

Supplement: Supplementary file 1 [file DataSheet_1.pdf]
